# A cross-sectional survey of English NHS Trusts on their uptake and provision of active bystander training including to address sexual harassment

**DOI:** 10.1177/20542704231166619

**Published:** 2023-05-04

**Authors:** Ava Robertson, Sarah Steele

**Affiliations:** 1 Population Health Sciences Partnership, Cambridge University, Cambridge, UK; 2 Cambridge Public Health, Forvie Site, 2152 University of Cambridge, Cambridge School of Clinical Medicine, Cambridge Biomedical Campus, Cambridge, UK; 3 Intellectual Forum, 61446 Jesus College, Cambridge, UK; 4 St Edmund's College, Cambridge, UK

**Keywords:** sexual harassment, NHS workforce training, active bystander training, continuing professional development

## Abstract

**Objective:**

Reports identify that sexual harassment is troublingly pervasive in the NHS. Active bystander training (ABT) has been promoted to address sexual harassment, alongside other forms of poor behaviour, discrimination, and harassment. We explore ABT across all English NHS Trusts and determine whether the programmes address sexual misconduct in the training content.

**Design:**

Freedom of Information requests asking whether Trusts offer ABT, and if so, about the programme content and delivery, and to NHS England on centrally commissioned ABT.

**Setting:**

213 NHS Trusts in England, and NHS England.

**Participants:**

Not applicable.

**Main Outcome Measure:**

Provision of ABT, and presence of sexual harassment content in the training.

**Results:**

199 Trusts responded by August 2022. Of these, 35 Trusts provide ABT, the majority of which deliver content that is not specific to sexual misconduct, are in London, outsource training using private providers, and only provide workshops on an opt-in basis. One Trust offers a standalone ABT module on sexual harassment. Private providers prohibit Trusts from sharing training material, inhibiting content analysis and evaluation. Among the 163 Trusts without ABT programmes, only 23 (13%) have plans to implement training.

**Conclusions:**

ABT is underutilised in the NHS, despite being identified as an effective intervention in other settings like the military, higher education, and government workplaces. Studies should explore whether wider NHS adoption is warranted. Robust monitoring and evaluation processes are critical to strengthening the available literature regarding the effectiveness of ABT in the healthcare context and engaging in global knowledge sharing across health systems.

## Introduction

Since 2017, when the #MeToo movement gained momentum around the world, sexual harassment in medicine has been extensively discussed, and recognised as both pervasive and harmful.^1^ Commonly understood as “improper behaviour that has a sexual dimension”, this form of harassment has major ramifications for the individuals it affects, as well as on societies, health systems and the healthcare workforce itself.^2–9^ While diversely conceived, this abuse—also referred to as ‘sexual misconduct’ by the GMC—^10^ is widely understood to include a range of verbal, online and physical acts, ranging from poor taste jokes to unwanted touching to rape.^11,12^

In the UK, NHS leadership reaffirmed their commitment to addressing workplace sexual harassment following a 2019 UNISON survey which indicated that 8 per cent of respondents have experienced it while at work during the last 12 months with at least 54 per cent of these acts being perpetrated by co-workers.^13–15^ Further impetus to respond was provided when the British Medical Association commissioned a report that detailed incidents of “lewd and inappropriate sexual remarks directed to, or made about women”, including “invitations or even instructions to accompany a male doctor to his hotel room”, something which took place at an annual professional meeting.^16,17^ Commitments to address such abuse have been numerous, and notably, calls have been made for prevention through interventions, as well as better responses when harassment is reported.^16,18^

Remarkably, despite such commitments, there is a dearth of research and clinical audits evaluating the effectiveness of interventions, especially within the healthcare setting. What data that do exist are largely from the United States, where legal requirements have been more robust, and where major employers like the Department of Defence have undertaken extensive trials of interventions, including, notably, Active Bystander Training (ABT).^19,20^ Indeed, within the US, ABT—a form of training that encourages individuals to recognise and respond to poor behaviour, by equipping people with skills to intervene through the usage of role-playing, case studies, and group participatory discussions that empower choices like taking direct action, delegation, distraction, delaying, and/or documenting—have been trialled around sexual harassment and then wound out, having been found to have positive results.^21,22^ A recent article identified the potential for use of this training within the NHS,, but raised the issue that there has been no examination of how widely ABT is being used, and what data we have on its efficacy.^12^

Indeed, to date, it is not known how widely ABT is being used across the NHS, whether any ABT deployed across the NHS includes content that addresses specifically sexual harassment alongside other issues like racism, homophobia, and other forms of discriminatory behaviour and abuse, or if any data could support curriculum development for wider training across the health service, profession, and medical schools. As such, here we use Freedom of Information requests with an aim to: (1) determine the number of English NHS trusts which provide ABT that contains training on sexual harassment and workplace misconduct; (2) ascertain whether training is made mandatory or elective, developed in-house or through an external provider; and (3) explore how trusts are delivering content through a content analysis of materials used in programmes to delineate similarities and differences in content.

## Methods

Requests for data were made to 213 NHS trusts in England listed on the NHS website in December 2021. According to the Freedom of Information Act 2000, an act of parliament that allows individuals to access information from public authorities, trusts had 20 working days to respond, either providing the data they hold or refusing the request according to grounds listed in the Act. Reasons for exemptions include that providing the data would violate an individual's right to privacy by requesting personal data; information is accessible via a publication scheme; answering the request is too costly of labour intensive (exceeding £450 or 18 staff hours for the NHS); disclosure of the information is against public interest or likely to result in prejudice; or the information held is part of a criminal investigation for example.^23^

The request acted as a cross-sectional survey, asking the trusts an initial closed-ended question to determine whether the NHS Trust provided ABT, specifically to their employees to address sexual harassment and misconduct in the workplace context. If yes, there was a series of follow-up questions which sought to determine whether this training was elective or mandatory, and a copy of the training materials was requested for qualitative analysis. If no, trusts were asked if there are plans to implement training. The use of a “yes” or “no” format enabled descriptive statistical analysis, while follow-up questions permitted trusts to provide additional qualitative data that the trust deemed relevant to the inquiry and to provide additional detail that the trust wished to include. Data were collected and responses were recorded using Microsoft Excel.

Subsequently, all pdf materials and written prose regarding interventions which the trusts included in their responses were analysed thematically to identify if any of the material overlapped with ABT. This was done to ensure the integrity of the study and avoid misclassification bias. For example, if the training materials satisfied the inclusion criteria and delivered content in keeping with the skills transfer expected with ABT, then the trust would be analysed as an ABT provider, even if it misclassified itself as not providing ABT.

## Results

### Response to the FOI

By August 2022, 93 per cent (*N* = 199) of Trusts responded, with 14 trusts failing to comply with the legislation, some citing COVID-related staffing issues and others simply not responding at all. One trust applied a Section 12 exemption stating it would “take more than 18 h to complete the request,” and was excluded from subsequent analysis. This indicates the Trust fails to maintain records of staff training in an accessible manner; a concerning suggestion from both the staffing and the Equality, Diversity, and Inclusion perspectives.

### Provision of training

Only 35 Trusts currently provide ABT, with 163 Trusts identified that they did not provide ABT at the time of the study. Of the 35 Trusts providing ABT, only 5 trusts identified that this training addresses sexual harassment in some form. The remaining 30 trusts identified that their training teaches participants to challenge antisocial behaviour only in a general context. Only one trust delivered content that specifically tackles sexual harassment in the workplace as its focus.

### Delivery of training

Quite importantly, one trust offering directed ABT around sexual harassment did so through a package of e-learning modules produced by a private provider, which had been newly added to their Learner Management System (LMS) and Electronic Staff Records. The Trust reported that this package included modules on: Sexual Harassment at Work; Understanding and Confronting Sexual Harassment; and The Effective Bystander. The Trust noted no staff members had taken the training at the time of its response, as it was “in the process of advertising it to Trust staff.”

Such use of private providers was notable across the responses. Only five trusts identified producing in-house training, while three trusts did not provide data and 27 Trusts identified outsourcing their training to one of three private companies which provide ABT to NHS Trusts: The Active Bystander Company, Skills Boosters, and Enact Solutions. Two trusts utilise Skills Boosters training and one trust utilises the services of Enact Solution, while the remaining 24 NHS trusts in this subset (22 of which are in London) provide ABT through the Active Bystander Company, which offers workshops it states are “applicable to all inappropriate behaviours including bullying, disablism, homophobia, racism, sexual harassment and transphobia” (Table 1). We note that Trusts did not identify that the content focused on sexual harassment, instead suggesting general content only, but because the training was provided by a private provider, and thus commercial in confidence, they were not able to supply learning materials or content.

### London trusts

Indeed, all London trusts which provide ABT are serviced by the Active Bystander Training Company, under training was commissioned by the NHS England (NHSE) as part of the London Workforce Race Strategy. This was undertaken because the London trusts provide services for large Black, Minority and Ethnic (BME) populations that have identified racial discriminatory behaviours. Several trusts confirmed that the training is at no cost to the trust itself. Training workshops were conducted on an elective basis and several trusts indicated that these workshops were delivered remotely via online platforms. One trust stated that all London *trusts “*could access up to 5 workshops free over the course of a year”. The training sessions (commissioned by the NHS) have a maximum capacity of 40 per session, this was confirmed in the results of the FOI request sent to NHSE stating that they commissioned ABT for “36 Trusts in London, along with other London-based NHS organisations”.

Despite NHSE budgeting training for 36 Trusts, only 22 Trusts in London utilised ABT at the time of the study. Of the remaining 14 Trusts that do not provide ABT, two identified they are in the process of scheduling sessions for 2022, one disclosed it had refused the offer, and the others provided no detail. Also, two London trusts declared that their staff initially received ABT funded by the NHSE but that the additional training is now funded by the Trust itself. One of these Trusts revealed plans to offer two training sessions per month to staff, a total of 24 sessions planned for 2022. Notably, Trusts paying for the programme directly could choose to increase session sizes beyond 40 delegates, but it is advised that “larger delegate numbers… should be regarded more as a ‘talk’ than a training session”.

### Content of training

Notably, Trusts were legally unable to provide the materials used as it infringes the copyright of private providers as the Trusts do not own the training content. Furthermore, Trusts which developed in-house training stated that the materials were unable to be provided as the material is stored in a format (such as an animation), that cannot be shared via email. However, several trusts attached the advertisement for the ABT Company which advertised training interventions summarised by 4D's: Direct action, Delegation, Distraction and Delay. These sessions are offered in either 60-, 75- and 90-min sessions and are interactive and include case-based scenarios and role-plays and delegates receive a “toolkit” at the end of the session. Considering the majority of trusts utilise the same provider, it is reasonable to assume the content and delivery across this subset of London trusts is relatively uniform.

### Plans to implement ABT

Among the 164 trusts not offering ABT, only 23 Trusts have active plans to implement it in the future. One trust stated that they are actively developing plans to develop sexual safety training which will incorporate ABT. They also stated that:

*It is (their) intention to develop future metrics to measure completion of training, and what difference it makes to both colleagues and people who use services, concerning sexual safety. Co-production will be an important element.*

Concerningly, several Trusts suggested they would consider implementing if there is, to quote one of these trusts, a “need for this form of training”, while other Trusts suggested implementation would occur if members of staff or working groups within the Trusts’ organisation advocate for it.

## Discussion

Our evidence reveals that the provision of ABT to address sexual harassment is inconsistent in NHS Trusts, and low across England, except in London, where most of the Trusts offering training are located, but even then, this training is not focused on sexual harassment. Most ABT programmes address undesirable behaviour and harassment in a general way only. This is deeply concerning considering the continued prevalence of sexual harassment in the healthcare sector and the staunch support of ABT by gender-based violence experts to reduce and prevent it. Only one Trust offered a package of online training modules directed to address sexual harassment, that included, rather commendably, ABT content.

While the generalisation of ABT content is beneficial in highlighting how to respond to broader poor behaviours including harassment and other forms of discrimination, previous literature advises caution in the adoption of “one-size fits all” approaches.^24,25^ The NHSE commissioned programmes were contracted to address racial discrimination, with the hopes of eliminating wider discriminatory behaviours, including sexual harassment, as a co-benefit. Merely having ABT is insufficient, the quality of the content is of paramount importance, especially when a multipronged approach, to ensure all issues are dealt with adequately.

Critically, we were unable to undertake such content assessment or to analyse data on behaviour change or feedback, as ABT provision in English NHS trusts is largely conducted by private, third-party providers. The NHS’ reliance on external providers to deliver critical professional development for the healthcare workforce is profound and has several implications. First, content ownership resides with the programme providers and is commercial in confidence. Consequently, training material cannot be externally audited, and best practice cannot be devised. This imposes serious limitations on training evaluation and examination of programme effectiveness.

Second, outsourcing ABT impedes knowledge transfer amongst employees within a trust, between NHS trusts, and amongst international colleagues.^26^ Attendees of the Active Bystander Training Company's workshops are said to receive a toolkit after the session which they are prohibited from sharing with other colleagues who did not attend. Furthermore, without access to training content, there is no informing best practice outside the Trust or in other health systems. While of value certainly to those in the session, knowledge is not more widely disseminated, and content is not shareable amongst the profession.

Thirdly, there are cost implications of the private provider model. The NHSE has budgeted £84,000.00 for ABT across all London Trusts and each trust can access up to 5 workshops. At the end of this initiative, it is expected to reach a total of 7640 staff members thus making the cost per person approximately £11.00 for a one-off trial. Notably, NICE does not have a cost threshold for sexual harassment interventions and has broadly endorsed even marginally effective prevention programmes as cost-effective, given the socioeconomic impacts of gender-based violence including sexual harassment. Nevertheless, it is still critical to consider whether ABT programmes would be more cost-effective if developed by Health Education England (HEE) and launched across their E-Learning for Health Care platforms. HEE is charged with the clinical and non-clinical training and skills development of the healthcare workforce. Content utilised in HEE training is delivered and provided, in consultation with the NHS. There are multiple advantages potentially gained from providing ABT under a centralised, public platform like improving access, enabling close monitoring and adaptation of curriculum, and facilitating research to inform best practice. In sum, the use of private companies to deliver human resource training has repercussions on learning across the NHS, on data collection, on monitoring and understanding of behaviour change around these issues from these types of programmes. The ethical, legal, and political dimensions of such outsourcing are outside the scope of this study but may be a point of discussion for future researchers.

Indeed, there are several limitations to this study. As with all studies using FOI requests, there is a potential for misinterpretation of the responses.^27–29^ Furthermore, the data requested is subject to mediation as the information officer will need to (1) determine which department is likely to hold information regarding staff training and (2) liaise with the relevant department to retrieve the desired data.^28,29^ This may have been the case in this study where some trusts answered the request stating that they provided Active Bystander Training, however upon examination of the documents and training description, the training may mention the role of bystanders around freedom to speak up and safeguarding but does not impart any skills to intervene. This result could indicate a lack of awareness of different types of sexual harassment training and interventions. However, despite the FOI being carefully crafted to ask targeted questions and extract precise information, one must contemplate that lack of clarity in the wording of the original FOI request as another explanation. Indeed, it is widely acknowledged that the utility of FOI requests in data collection hinges on the careful selection of terminology used.^29^ For this reason, information officers were encouraged to seek clarification to avoid unnecessary assumptions and all documents sent by the Trusts were examined against the inclusion criteria regardless of the Trust's response to the yes/no question.

However, the strength of this study is that it has given insight into the current provision of ABT and raises serious concerns regarding training content provided due to the inaccessibility of material for external audit. Ideally, assessments should be conducted before and post-intervention,^20^ and the use of private providers for training have meant that such data are not publicly available, nor are learning materials. Notably, NHS sites which do not currently provide ABT are optimal sites for future intervention, utilising content that addresses sexual harassment specifically, encouraging direct intervention, and focuses on teaching participants to recognise socially risky scenarios that warrant attention. This presents an opportunity to pilot ABT material that is not commercial in confidence and thus enables public health data collection, external monitoring and evaluation frameworks, and dissemination of knowledge.

Concerningly, in their responses, several trusts briefly mentioned the suspension of certain training modules, including Equality Diversity and Inclusion, Safeguarding or Bullying and Harassment, during the pandemic. This presents an opportunity for further health systems-based research to delineate whether this was a common trend across all trusts, whether there is a correlation between cessation of training based on geographic location or type of trust, the average duration of this cessation, and whether training cessation was common to all human resource training programmes or specific to EDI- and safeguarding-type training. Indeed, it is understandable that the healthcare sector operated at reduced capacity and was forced to divert resources as part of its pandemic response. However, research notes sexual harassment and workplace misconduct worsen during humanitarian crises and high-stress situations,^29^ hence, continued training, inclusive of modules which promote prosocial behaviour, safeguarding, respect, and inclusivity is critical. Research on this may feed into future pandemic planning.

## Conclusion

In line with our observations, we call upon NHS leaders, Clinical Commissioning Groups, and policymakers to recognise the issues around training, including but not limited to the outsourcing of vital training to private providers. Without access to training materials for external evaluation, there is no means to assess the training adequacy and to share knowledge across NHS bodies. With only a minority of trusts offering any ABT, and only one Trust offering ABT around sexual harassment, evaluation and knowledge sharing are critical. While tenders and utilisation of private companies to perform public acts are meant to increase competition, it is evident that regarding ABT, one provider dominates. Policymakers should heed caution before winding out further training and recall that private companies have commercial interests to protect. Therefore, it would be prudent to utilise the existing, public frameworks to provide sexual harassment and ABT in the future.

Notably, outside the medical world, there is now active piloting of ABT interventions to reduce sexual harassment and violence against women and girls, including a national campaign in the UK run by the Home Office. Cross-departmental learning from this Home Office campaign, which seeks to effect behaviour change and encourage active intervention on a range of forms of sexual harassment and abuse, would aid the Department of Health and Social Care, and the NHS, in thinking about behaviour change interventions amongst healthcare staff. What is clear is that it is incumbent on healthcare leaders, policymakers, and professional organisations to encourage sexual harassment training from the very first days of undergraduate degrees through to the day of retirement. A core priority for the future, therefore, will be to ensure that training content on sexual harassment is adaptable to different NHS contexts and changing needs, not over burdensome to staff, and that can realistically be delivered in a general healthcare setting to all healthcare workers.

**Table 1.** Provision of active bystander training by region.

NHS Region	Responded Yes n (%)	Responded No n (%)	No Response n (%)	Total n (%)
East of England	4 (11)	16 (10)	2 (14)	22 (10)
London	22 (62)	12 (7)	2 (14)	36 (17)
Midlands	3 (9)	34 (21)	4 (30)	41 (19)
Northeast and Yorkshire	1 (3)	28 (17)	3 (21)	32 (15)
Northwest	1 (3)	29 (18)	1 (7)	31^a^ (15)
Southeast	3 (9)	24 (15)	2 (14)	29 (14)
Southwest	1 (3)	20 (12)	0 (0)	21 (10)
Total (n)	35	163	14	212 (100)
NHS Region	Responded Yes n (%)	Responded No n (%)	No Response n (%)	Total n (%)
Non-London	13 (38)	151 (93)	12 (86)	176 (83)
London	22 (62)	12 (7)	2 (14)	36 (17)
Total (n)	35	163	14	212 (100)

^a^ One trust applied a section 12 exemption and was excluded from the analysis.

**Declarations**
